# HIV-Associated Microstructural Abnormalities in Default Mode, Executive Control, and Salience Networks: Insights from Tensor-Valued Diffusion Encoding

**DOI:** 10.3390/bioengineering13040413

**Published:** 2026-04-01

**Authors:** Md Nasir Uddin, Abrar Faiyaz, Chase R. Figley, Xing Qiu, Miriam T. Weber, Giovanni Schifitto

**Affiliations:** 1 Department of Neurology, University of Rochester, Rochester, NY 14642, USA; abrar_faiyaz@urmc.rochester.edu (A.F.); miriam_weber@urmc.rochester.edu (M.T.W.); giovanni_schifitto@urmc.rochester.edu (G.S.); 2 Department of Biomedical Engineering, University of Rochester, Rochester, NY 14627, USA; 3 Department of Electrical and Computer Engineering, University of Rochester, Rochester, NY 14627, USA; 4 Department of Radiology, University of Manitoba, Winnipeg, MB R3A 1R9, Canada; chase.figley@umanitoba.ca; 5 Department of Biostatistics and Computational Biology, University of Rochester, Rochester, NY 14642, USA; xing_qiu@urmc.rochester.edu; 6 Department of Imaging Sciences, University of Rochester, Rochester, NY 14642, USA

**Keywords:** tensor-valued diffusion MRI, HIV, brain, neuroimaging, default mode network, executive control network, salience network, cognition

## Abstract

Cognitive impairment persists in people with HIV (PWH) despite effective combination antiretroviral therapy, possibly as a result of persistent alterations in white matter microstructural abnormalities in the brain. Noninvasive tensor-valued diffusion MRI (dMRI) is sensitive to microstructural integrity; thus, it may contribute to the understanding of HIV-associated cognitive impairment. In this exploratory cross-sectional study, 31 healthy controls (HCs) and 24 PWH underwent 3T MRI and neurocognitive assessment. Tensor-valued dMRI metrics, including microscopic fractional anisotropy (µFA) and isotropic, anisotropic, and total mean kurtosis (MKi, MKa, MKt), and conventional DTI and DKI metrics (FA, MD, and MK) were evaluated across six functionally defined brain networks. Compared with HCs, PWH exhibited reduced FA, µFA, and MKa in the dorsal default mode and anterior salience networks, along with increased MKi in the salience network and decreased MKi in the executive control network, with moderate effect sizes. Compared with HCs, PWH performed significantly worse on measures of learning, memory, and language, but showed no differences in executive function, attention, or processing speed. Additionally, significant associations and interactions between dMRI metrics and HIV status were observed, particularly for MKi and attention, executive function, and processing speed across the default mode, salience, and executive control networks. These preliminary findings underscore tensor-valued dMRI as a sensitive biomarker of network-specific neurocognitive vulnerability in HIV.

## 1. Introduction

Although combination antiretroviral therapy (cART) has substantially improved clinical outcomes, people with HIV (PWH) continue to show a risk of cognitive impairment, with nearly 50% exhibiting subtle changes in executive function, information processing speed, attention, and motor skills, even with effective viral suppression [1,2,3]. Although combination antiretroviral therapy (cART) has substantially improved clinical outcomes, PWH continue to show a risk of cognitive impairments. These impairments are thought to arise from a combination of factors, including past uncontrolled viral replication, persistent immune activation, and cART-associated neurotoxicity [3]. Chronic mild neuroinflammation is considered a primary driver in HIV-associated cognitive impairment, and the aging population on cART faces additional risks from comorbidities and certain neurotoxic antiretroviral regimens [4,5,6]. Advanced, noninvasive, sensitive, and reproducible neuroimaging methods may help to understand mechanisms of neurocognitive dysfunction in HIV by better characterizing HIV-associated microstructural injury.

Diffusion magnetic resonance imaging (dMRI) provides a quantitative assessment of tissue microstructure noninvasively by sensitizing the MR signal to the stochastic displacement of water molecules [7,8,9,10,11]. In conventional diffusion tensor imaging (DTI), scalar indices such as mean diffusivity (MD) and fractional anisotropy (FA) are estimated to characterize average diffusivity and diffusion anisotropy within a voxel, respectively. However, the diffusion tensor model is constrained by a Gaussian diffusion assumption and a single-compartment representation, which limits its specificity in regions with complex fiber architecture, heterogeneous cellular environments, or increased extracellular water content [12,13,14]. Therefore, differences in microstructural characteristics, such as axonal density, fiber orientation dispersion, and partial-volume effects, may be confounded by DTI-derived metrics. Advanced dMRI models have been developed to better characterize pathological tissue and overcome these constraints. Diffusion kurtosis imaging (DKI), for example, offers enhanced sensitivity to microstructural heterogeneity and captures non-Gaussian diffusion behavior [15,16]. Previous works on HIV have shown that PWH have lower FA and mean kurtosis (MK) than HCs, which suggests that DKI may be able to identify subtle microstructural abnormalities [17]. However, because MK reflects several microstructural characteristics, including anisotropy, compartmental heterogeneity, and microscopic restrictions, which are combined into a single MK metric in standard DKI, limiting biological specificity, the biological interpretation of MK is still difficult.

By using multidimensional b-tensor encoding schemes, such as linear and spherical tensor encodings (LTE and STE), which more accurately capture the intricate microstructure of brain tissue, tensor-valued dMRI overcomes the aforementioned limitations [18]. The total mean kurtosis (MKt) can be decomposed into anisotropic (MKa) and isotropic (MKi) components using this framework. MKa predominantly reflects variance in diffusion arising from orientation-dependent microstructural anisotropy, such as axonal alignment and dispersion, whereas MKi quantifies orientation-invariant diffusion heterogeneity related to variability in compartmental size, cellular packing, and changes in intracellular volume fraction. In addition, unlike FA, which is strongly confounded by fiber orientation dispersion and crossing fibers [10], tensor-valued diffusion encoding decouples microscopic fractional anisotropy (µFA) from macroscopic orientation effects [19,20,21]. That is, tensor-valued dMRI provides more specific and biologically meaningful measures of tissue microstructural integrity. Several previous studies have documented that tensor-valued dMRI metrics have demonstrated increased sensitivity for detecting microstructural abnormalities in both gray and white matter in HIV and other neurological disorders, offering improved interpretability and performance compared with conventional DTI- and DKI-based measures [20,22,23].

It is well established that HIV is associated with white matter abnormalities that can lead to cognitive impairment [24,25,26]. However, the pathways through which HIV-related brain changes translate into deficits across cognitive domains remain incompletely characterized, and prior dMRI studies have reported inconsistent findings [27,28]. In addition, most prior MRI studies, particularly those using dMRI, have relied on anatomically defined regions of interest (ROIs) to extract imaging metrics and relate them to cognitive performance. However, cognitive functions are mediated by distributed, network-level circuits rather than strictly defined anatomical regions. Functionally defined atlases [29,30,31] provide a more biologically meaningful framework by grouping voxels with similar functional connectivity or microstructural roles, thereby reducing within-ROI heterogeneity and partial-volume effects. This approach enhances sensitivity and specificity when linking microstructural abnormalities to cognitive outcomes, especially in conditions characterized by subtle, diffuse, or network-specific damage.

In this pilot study, we investigated conventional DTI and DKI, and tensor-valued dMRI metrics across functionally defined white matter brain networks and evaluated their associations with cognitive performance in PWH and HCs. We hypothesized that tensor-valued dMRI would reveal network-specific microstructural alterations in PWH and demonstrate stronger and more specific relationships with cognition than conventional DTI and DKI measures.

## 2. Materials and Methods

***Study Participants:*** A prior study reported quantitative dMRI analysis in gray and white matter using an anatomically defined atlas and examined their associations with cognition and blood markers [32]. The present work analyzed the same dMRI dataset from the same cohort using functionally defined brain networks to assess relationships with cognitive performance. This work was conducted as a sub-study of a project approved by the Institutional Research Subjects Review Board (RSRB) at the University of Rochester. Twenty-four PWH (mean age 55 ± 10 years; 7 female) and 31 age- and sex-matched HC participants (mean age 55 ± 15 years; 7 female) were recruited from Rochester, NY, and surrounding areas. Written informed consent was obtained from each participant prior to their participation in the study. Participants then underwent a series of clinical, laboratory, neurocognitive, and brain MRI evaluations, all conducted in accordance with approved ethical and regulatory frameworks. A brief overview of cohort demographics and clinical characteristics is presented in Table 1. It is noteworthy that the study participants had several major comorbidities, including hypertension, hyperlipidemia, high cholesterol, anxiety, depression, and neuropathy.

Detailed inclusion and exclusion criteria have been reported previously [33]. Briefly, PWH were ≥18 years old, on stable cART for at least three months before screening, and HIV-1 seropositive with documented infection, and had viral load ≤200 copies/mL. Exclusion criteria included symptomatic cardiovascular disease, uncontrolled vascular risk factors, severe psychiatric disorders, non-HIV-related brain infections, space-occupying lesions, dementia, and MRI contraindications. The control group differed (*p* < 0.05) from the PWH group by HIV status, education level, and race.

***Neuropsychological assessments:*** All participants completed a comprehensive neuropsychological battery assessing seven cognitive domains. Information processing speed was evaluated with the Symbol Digit Modalities Test, the Stroop Color and Word Test (Color Naming), and the Trailmaking Test A. Executive function was evaluated using the Stroop Color and Word Test (Interference) and the Trailmaking Test B, attention and working memory were evaluated via the Letter Number Sequencing subtest of the Wechsler Adult Intelligence Scale and the California Computerized Assessment Package (CalCAP), learning was evaluated with the Immediate recall trials of the Rey Auditory Verbal Learning Test and the Rey Complex Figure Test, memory was evaluated with the delayed recall portions of both tests, language was evaluated with the Controlled Oral Word Association Test, and motor function was evaluated with the Grooved Pegboard test.

All tests were scored and administered by trained psychometrists under the supervision of a licensed clinical neuropsychologist. For each individual test, raw scores were converted to standardized z-scores using published, manualized norms that account for age and education. Composite domain scores were then computed by averaging the z-scores of the constituent tests within each of the seven domains. A total cognitive z-score was also calculated by summing the seven domain-specific z-scores.

***MRI data acquisition:*** MRI data were acquired on a 3T whole-body scanner (MAGNETOM Prisma Fit, Siemens Healthineers, Erlangen, Germany; software version VE11c) equipped with a 64-channel head coil. The system provides a maximum gradient strength of 80 mT/m and a slew rate of 200 mT/m/s.

Anatomical MRI: High-resolution anatomical images were obtained using a three-dimensional T1-weighted (T1w) magnetization-prepared rapid acquisition gradient-echo (MPRAGE) sequence. Imaging parameters included inversion time (TI) = 926 ms, repetition time (TR) = 1840 ms, echo time (TE) = 2.45 ms, GeneRalized Autocalibrating Partial Parallel Acquisition (GRAPPA) acceleration factor = 2, and flip angle = 8°, sagittal orientation, field of view (FOV) = 256 × 256 × 192 mm^3^, total number of slices = 192, resolution = 1.0 × 1.0 × 1.0 mm^3^, and total scan time = 4 min 26 s.

Tensor-valued diffusion encoding: Diffusion-weighted imaging (DWI) was performed using a prototype free-waveform (FWF) encoding single-shot spin-echo sequence with concomitant gradient compensation (v1.13s) [34]. The sequence implementation is publicly available (https://github.com/filip-szczepankiewicz/fwf_seq_resources, accessed on 5 September 2023). Eighty DWI volumes were acquired, consisting of 43 volumes with linear tensor encoding (LTE) and 37 volumes with spherical tensor encoding (STE). Data were sampled at multiple diffusion weightings (b = 0, 100, 700, 1400, and 2000 s/mm^2^). Sequence parameters included TR = 4100 ms, TE = 91 ms, FOV = 224 × 224 × 30 mm^3^, echo spacing = 0.6 ms, GRAPPA acceleration factor = 2, partial Fourier acquisition = 6/8, axial orientation, number of slices = 30, and resolution = 2.0 × 2.0 × 4.0 mm^3^. Phase encoding was applied in the anterior–posterior direction, and total scan time was 5 min 53 s.

***Image Analysis:*** Image analyses were performed using MATLAB (version 2023b), FMRIB Software Library (FSL https://fsl.fmrib.ox.ac.uk/fsl/fslwiki/, accessed on 5 September 2023) [35], and Advanced Normalization Tools (ANTs http://stnava.github.io/ANTs/, accessed on 5 September 2023) [36].

All images were visually checked to identify major artifacts, such as subject bulk motion, geometric distortion, or signal loss. T1w anatomical images were then processed using FSL’s fsl_anat pipeline [37], which included image reorientation, correction of intensity nonuniformity, skull stripping using Brain Extraction Tool (BET) [38], tissue segmentation with FMRIB’s Automated Segmentation Tool (FAST), subcortical segmentation via FMRIB’s Integrated Registration and Segmentation Tool (FIRST), and both linear and nonlinear registration to the MNI 2 mm template using FMRIB’s Linear Image Registration Tool (FLIRT) and FMRIB’s Nonlinear Image Registration Tool (FNIRT).

The dMRI metrics (b-tensor encoded, DTI, and DKI) were quantified using a multidimensional dMRI (MD-dMRI) framework [39] (https://github.com/markus-nilsson/md-dmri, accessed on 5 September 2023). Major image processing steps: (1) raw DWI images were corrected for eddy-current-induced distortions and inter-volume subject motion using an extrapolation-based registration implemented in ElastiX (version 5.0.0) [40,41], which is particularly suited for datasets acquired at high diffusion weightings; (2) the images were spatially smoothed using a 3D Gaussian filter with a standard deviation of 0.4 voxels; (3) voxel-wise dMRI metrics were estimated by fitting the log-transformed signal using linear least-squares regression with heteroscedasticity correction via the *dtd_covariance* method [19,21,39]. This procedure yielded measures of anisotropic, isotropic, and total diffusional variance (MKa, MKi, MKt) and µFA. Conventional DTI metrics, including FA and MD, as well as DKI metrics such as MK, were also computed from the same tensor-valued dMRI dataset using the same processing framework [42].

**Figure 1:**
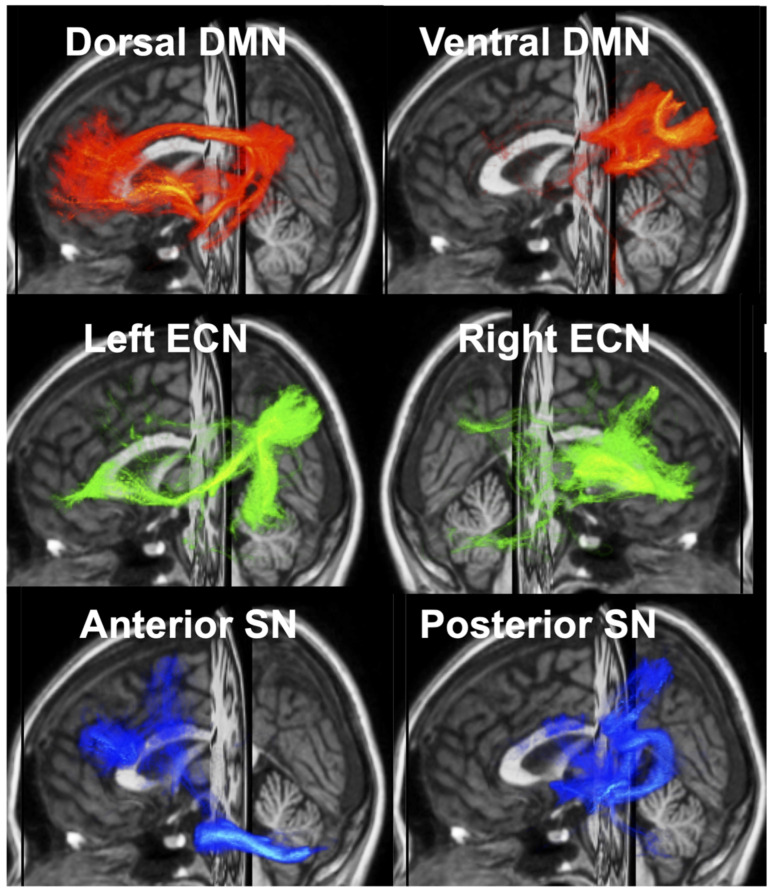
3D depictions of the six functionally defined human white matter networks investigated in the current study overlaid on the T1-weighted MNI152 template: Dorsal and ventral Default Mode Network (DMN_D, DMN_V), left and right Executive Control Network (ECN_L, ECN_R), anterior and posterior Salience Network (SN_A, SN_P).

**ROI analysis:** The UManitoba–JHU functionally defined human white matter atlases [29,30] and b-tensor derived maps were nonlinearly registered to native DWI space using ANTs [43]. ROI mean values were then extracted from white matter pathways corresponding to 6 previously defined intrinsic functional brain networks [31], including the dorsal and ventral Default Mode Network (DMN_D, DMN_V), left and right Executive Control Network (ECN_L, ECN_R), and anterior and posterior Salience Network (SN_A, SN_P). Although the atlas defines 12 functional networks, we focused our analyses on these 6 networks due to their established involvement in HIV-associated cognitive impairment [44,45]. Figure 1 illustrates the 6 functionally defined brain networks.

**Statistical Analyses:** All statistical analyses were performed using Python (version 3.7.4). with NumPy (version 1.26.3) and SciPy (version 1.11.4), and data visualization was conducted using Seaborn (version 0.11.2). Group differences in dMRI metrics within functionally defined ROIs were evaluated using two-sample *t*-tests. Associations between dMRI metrics and cognitive performance were assessed using Spearman correlation analyses with age and sex included as covariates to control for their potential confounding effects.

To examine the combined effects of HIV status and dMRI metrics on cognitive performance, an ordinary least squares (OLS) linear regression model was constructed with cognitive performance modeled as a function of dMRI metrics, HIV status, and their interaction (i.e., Cognitive score = dMRI metrics + HIV status + dMRI metrics × HIV status). Statistical significance was defined as *p* < 0.05 for single comparisons.

Effect sizes were calculated to quantify the magnitude of observed associations and group differences. For group comparisons, Cohen’s *d* was computed from group means and standard deviations using the pooled standard deviation. For correlation analyses, effect sizes were derived from Spearman correlation coefficients (*r*) using the transformation ($d=\frac{2r}{\sqrt{1-r^{2}}}$). For OLS linear regression models comparing two groups with interactions, Cohen’s *d* was estimated from the *t*-statistic using ($d=t\sqrt{\frac{1}{n_{1}}+\frac{1}{n_{2}}}$, where *n*_1_ and *n*_2_ represent the sample sizes of the two groups. Effect sizes were interpreted using conventional benchmarks (small ≈ 0.2, medium ≈ 0.5, large ≥ 0.8).

## 3. Results

**Figure 2:**
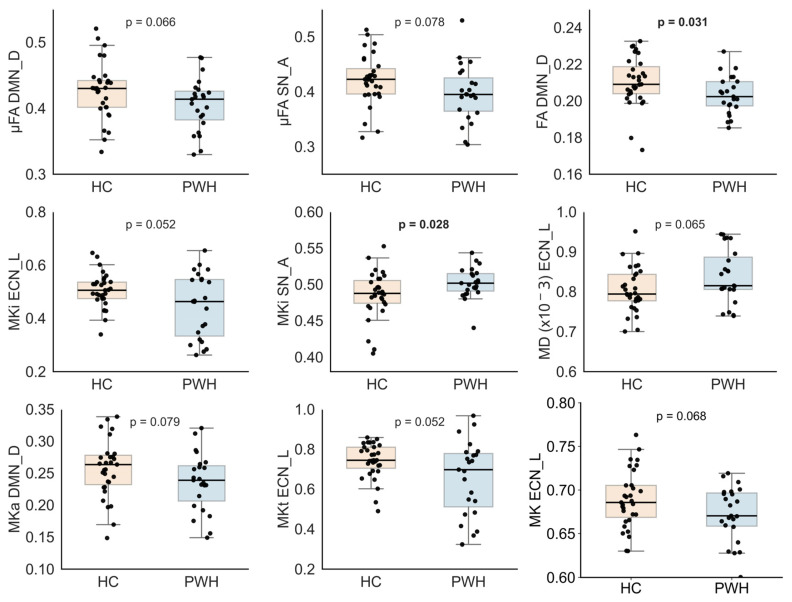
Comparison of diffusion MRI (dMRI) metrics across functionally defined brain networks (*p* < 0.1). Statistically significant uncorrected results (*p* < 0.05) are shown in bold; tensor-valued dMRI metrics include microscopic fractional anisotropy (μFA), isotropic mean kurtosis (MKi), anisotropic mean kurtosis (MKa), and total mean kurtosis (MKt). DTI metrics include fractional anisotropy (FA) and mean diffusivity (MD), and DKI-derived mean kurtosis (MK). Brain networks include the dorsal default mode network (DMN_D), anterior salience network (SN_A), and left executive control network (ECN_L). PWH denotes people with HIV, and HC denotes healthy controls.

Figure 2 illustrates group comparisons of dMRI metrics across functionally defined brain networks, including only regions showing effects at *p* < 0.1. We found that PWH demonstrated evidence of altered tissue microstructure relative to HCs, particularly within core cognitive networks. As expected, both µFA and conventional FA, as well as MKa, were reduced in the DMN_D and the SN_A in PWH compared with HCs. A statistically significant reduction was observed for FA in DMN_D (*p* = 0.031), while reductions in µFA and MKa in DMN_D and SN_A reached marginal significance (*p* > 0.05 but <0.1).

In contrast, PWH revealed significantly increased MKi in SN_A (*p* = 0.028), whereas MKi was reduced in the ECN_L (*p* = 0.052) compared with HCs. Additionally, MKa was marginally decreased in DMN_D (*p* = 0.079), MKt was reduced in DMN_V (*p* = 0.074) and ECN_L (*p* = 0.052), and MK was reduced in ECN_L (*p* = 0.068) in PWH relative to HCs.

**Figure 3:**
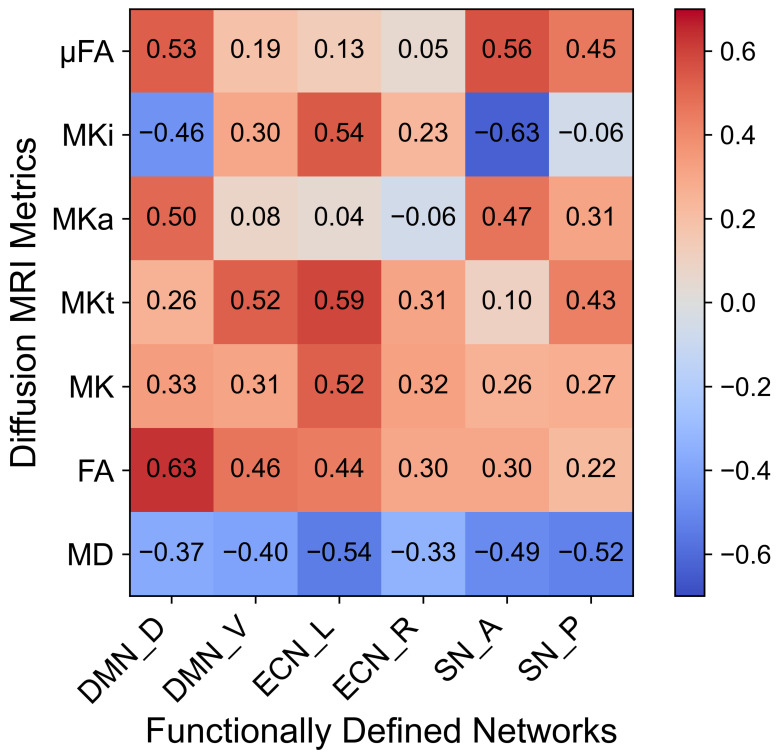
Heatmap of effect sizes (Cohen’s d) for diffusion MRI metrics across six functional brain networks. Positive values indicate higher values in HCs relative to PWH, while negative values indicate higher values in PWH.

Further, the effect size analysis revealed small to moderate group differences across dMRI metrics and functional networks (Figure 3). FA had the strongest positive effects in the DMN_D (*d* = 0.63) and MKt in the ECN_L (*d* = 0.59), while MD had consistently negative effects across networks (*d* ≈ −0.33 to −0.54). MKi in SN_A had a significant negative effect (*d* = −0.63), suggesting network-specific microstructural differences between HCs and PWH.

**Figure 4:**
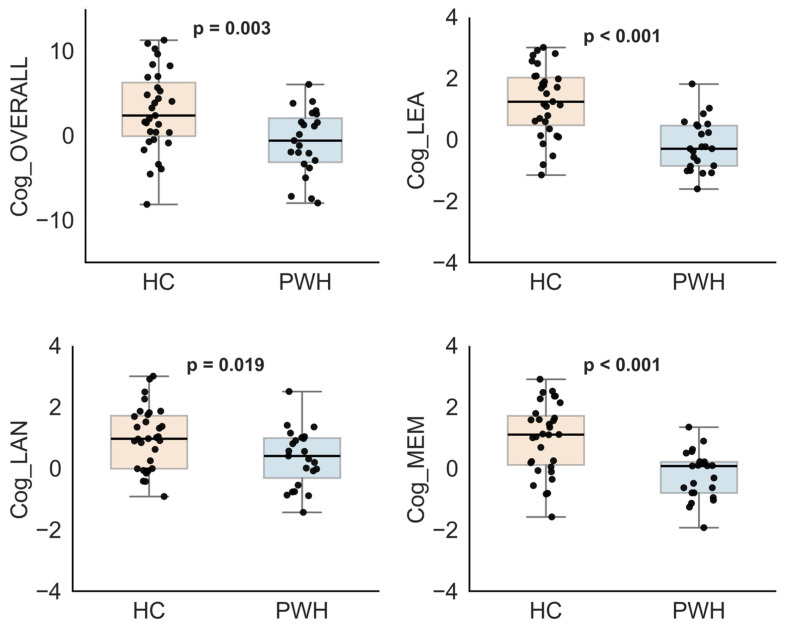
Comparison of cognitive scores between healthy controls (HCs) and people with HIV (PWH) (*p* < 0.05). Both overall and domain-specific cognitive scores are consistently higher in HCs than in PWH. Significant group differences were observed for the total cognitive score (Cog_OVERALL), learning (Cog_LEA), language (Cog_LAN), and memory (Cog_MEM) domain scores.

Figure 4 presents comparisons of cognitive domain scores between PWH and HCs. PWH had significantly lower overall cognitive scores than HCs. Follow-up analyses revealed that these differences were specific to the learning, language, and memory domains (*p* < 0.05). In contrast, no significant group differences were observed for executive function, attention, motor skills, or processing speed. In addition, effect size analysis indicated large group differences in learning (*d* = 1.43) and memory (*d* = 1.22), as well as moderate to large effects in overall cognition (*d* = 0.84) and language (*d* = 0.66). Executive function, attention, and processing speed domains, on the other hand, showed only small effects.

We next examined associations between cognitive scores and tensor-valued dMRI metrics across six functionally defined brain networks. Spearman correlation coefficients and effect sizes between dMRI metrics and cognitive domains are summarized in Table 2. In PWH, most significant correlations (|*r*| ≈ 0.5, *p* < 0.05) involved executive function (EXE), which was associated with µFA and MKa (positive) and MKi and MD (negative) in DMN_D, DMN_V, ECN_R, and SN_A, with large effect sizes (|*d*| ≈ 0.9–1.35). Processing speed (SPE) also showed significant associations with MKi across multiple networks, including DMN_D, DMN_V, and SN_A (|*r*| ≈ 0.6, *p* < 0.05, |*d*| up to 1.58). In contrast, among HCs, significant correlations were predominantly observed between attention (ATT) and µFA, MKa, MKt, and MD within several networks, including DMN_D, DMN_V, ECN_R, and SN_A (*p* < 0.05), with large effect sizes (|*d*| ≈ 0.9–1.28).

**Figure 5:**
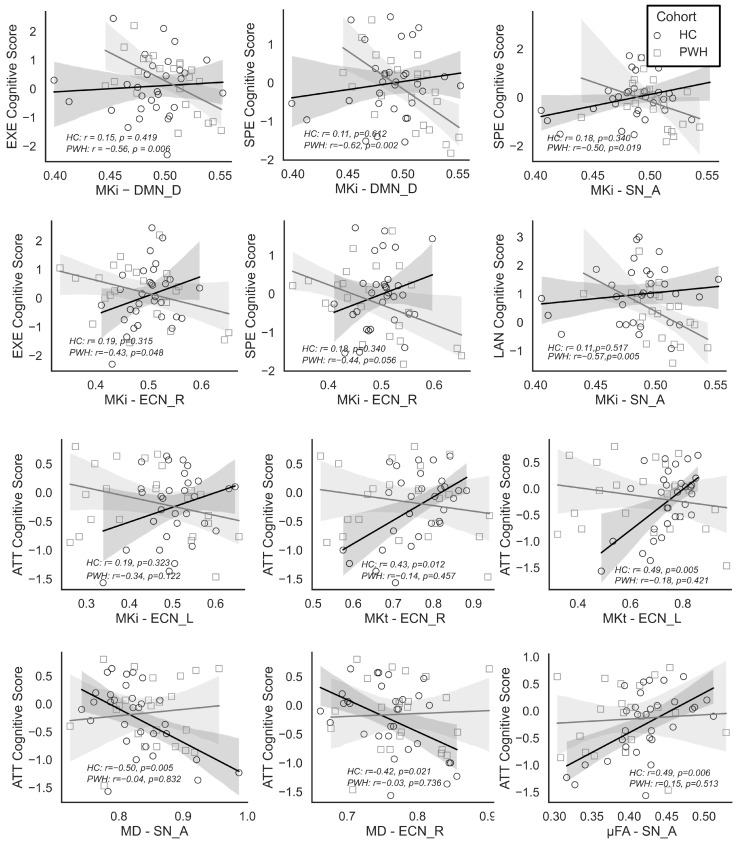
Relationships between cognitive domain scores and diffusion MRI (dMRI) metrics (b-tensor encoding and DTI) within functionally defined brain networks showing significant interactions with HIV status (*p* < 0.05). Cognitive domains include executive function (EXE), information processing speed (SPE), language (LAN), and attention/working memory (ATT). Diffusion metrics include isotropic mean kurtosis (MKi), total mean kurtosis (MKt), fractional anisotropy (FA), microscopic fractional anisotropy (uFA), and mean diffusivity (MD). PWH denotes people with HIV, and HC denotes healthy controls.

We used an ordinary least squares (OLS) linear regression model to further investigate associations between cognitive domain scores and dMRI metrics, assessing the main effects of HIV status and dMRI metrics, as well as their interaction, across six functionally defined ROIs. Consistent with our two-group analyses, HIV status exhibited significant main effects on cognitive scores across all reported models. While dMRI metrics did not show significant main effects, their interactions with HIV status were significant across multiple networks, strongly suggesting that there are HIV-specific associations between dMRI measures and cognition. Figure 5 depicts the plots of cognitive scores versus dMRI measures, whereas Table 3 summarizes representative results, reporting only statistically significant interactions.

In DMN_D, executive function (EXE) interacted significantly with MKi (*d* = −0.65, *p* = 0.018), and HIV status had a significant main effect (*d* = 0.67, *p* = 0.016). EXE also showed a significant interaction (*d* = −0.59, *p* = 0.031) and a main effect of HIV status (*d* = 0.60, *p* = 0.029) in the ECN_R.

For attention (ATT), MKi in the ECN_L had a significant interaction with HIV status (*d* = −0.57, *p* = 0.037). MKt had stronger effects in both ECN_L (*d* = −0.94, *p* = 0.001) and ECN_R (*d* = −0.82, *p* = 0.004) and showed significant interactions with HIV status. In addition, MD in the ECN_R (*d* = 0.54, *p* = 0.047) and MD in the SN_A (*d* = 0.65, *p* = 0.019) had significant interaction effects. Significant interactions with medium effect sizes were also found for µFA (*d* = −0.55, *p* = 0.044) and FA (*d* = −0.63, *p* = 0.021) in the SN_A.

HIV status significantly influenced processing speed (SPE) across several networks (*d* = 0.59–0.82, *p* < 0.05). MKi showed significant interaction effects in the DMN_D (*d* = −0.83, *p* = 0.003), ECN_R (*d* = −0.62, *p* = 0.024), and SN_A (*d* = −0.70, *p* = 0.012). Furthermore, a significant interaction between MKi and language (LAN) was found in SN_A (*d* = −0.65, *p* = 0.018).

## 4. Discussion

In this pilot study, we used dMRI to investigate the effects of HIV infection on white matter microstructure across functionally defined brain networks and how these microstructural changes relate to domain-specific cognitive performance. Our findings show that tensor-valued dMRI metrics are more sensitive to microstructural alterations in functionally defined networks than conventional DTI and DKI metrics, as evidenced by group differences in PWH and HCs. In addition, PWH exhibit significant associations and interactions between dMRI metrics, with tensor-valued metrics having a higher sensitivity to HIV status, which influences cognitive outcomes compared to HCs. These results demonstrate that tensor-valued dMRI metrics outperform conventional dMRI metrics in quantifying subtle neuroaxonal damage and the impact of HIV on cognition. It should be noted that the primary goal of this study is hypothesis generation in an exploratory pilot context; we present unadjusted *p*-values and effect sizes for each analysis.

We observed reductions in FA, µFA, and MKa in key functionally defined brain networks, including the DMN_D and SN_A, supporting prior evidence that HIV infection causes widespread white matter microstructural changes [46,47,48,49]. The significant reduction in FA in DMN_D and marginal reductions in related metrics are consistent with previous studies reporting lower FA and higher MD in white matter tracts of PWH, indicating compromised microstructural integrity of white matter tracts that support cognition [17,50]. According to recent meta-analyses, PWH consistently show lower FA and higher MD than HCs, suggesting diffuse white matter injury even in those receiving cART [51,52].

However, we found a significant increase in MKi in SN_A and a decrease in MKi in ECN_L. These diverse patterns of isotropic kurtosis indicate complex microstructural changes that vary across functionally defined brain networks and may result from a combination of processes, such as compensatory reorganization, axonal degeneration, and neuroinflammation. Multi-shell dMRI studies in PWH also show widespread involvement of white matter pathways involved in attention and memory domains, supporting the idea that advanced dMRI metrics can reveal subtler alterations than conventional DTI alone [17]. PWH had significantly lower cognitive function than HCs, especially on measures of learning, memory, and language. This is consistent with extensive neuropsychological evidence demonstrating that HIV infection is frequently associated with cognitive declines, particularly in episodic memory and complex cognitive processing, even during the cART era [53].

In contrast to previous studies [54,55,56], we found no significant group differences in executive function, attention, or processing speed. Our correlation analyses, however, revealed a significant association of tensor-valued dMRI metrics with cognitive domain scores, specifically executive function, and processing speed in PWH, as well as attention, for most of the b-tensor encoding metrics and MD. Effect size estimates revealed moderate-to-large correlations (|d| ≈ 0.8–1.6), indicating that microstructural alterations in functional brain networks, especially within the DMN, ECN, and SN, may influence variations in executive function, attention, and processing speed in PWH.

Furthermore, our interaction analyses revealed that HIV status significantly influences the relationship between microstructural integrity, primarily as measured by b-tensor encoding, and cognitive performance across several subdomains. Although interactions for overall cognitive scores did not reach statistical significance, these results suggest that tensor-valued dMRI metrics are sensitive to microstructural changes within brain networks that influence specific cognitive domains, particularly executive function, attention, and processing speed.

Executive function showed significant interactions with MKi in both the DMN_D and ECN_R, suggesting that microstructural changes in these networks disproportionately affect executive outcomes in PWH. These findings are consistent with functional connectivity studies that have shown that disruptions in the DMN and its coupling with executive networks are associated with declines in higher-order cognition in HIV-associated neurocognitive impairment [45,57].

The attention domain was highly sensitive to microstructural interactions across multiple networks, including significant effects of MKi, µFA, FA, and MD in ECN_R, ECN_L, and SN_A. This suggests that attention performance in PWH is highly dependent on the integrity of both executive control and salience networks, and that this may also be true in other situations. Prior research has shown that the salience and executive control networks play an important role in attentional control and cognitive resource allocation, and that disruptions in these networks are associated with attentional deficits in PWH [45,58]. The prominence of attention in the interaction results suggests that it is especially vulnerable to early network compromise.

Processing speed also showed significant interactions with MKi in DMN_D, ECN_R, and SN_A, indicating that the microstructural complexity of these networks modulates cognitive processing speed differently in PWH. Processing speed is frequently affected in HIV-associated cognitive impairment, and it is regarded as one of the sensitive cognitive domains to be influenced by microstructural brain changes [59,60]. Advanced dMRI studies have linked variations in white matter integrity to slower information processing across clinical populations [61,62,63], implying that tensor-valued dMRI metrics capture microstructural features that support efficient neural communication and cognitive function.

Furthermore, domain-specific interactions were observed for learning in DMN_D and for language in SN_A, suggesting that microstructural integrity within memory and language networks influences these cognitive processes in PWH. These network-dependent effects highlight the benefit of looking at dMRI metrics within functionally defined brain regions rather than the entire brain, as this approach may reveal more specific structure-function relationships related to HIV-associated neurocognitive impairment [45]. Notably, DKI-derived MK and DTI-derived FA were insensitive markers for distinguishing PWH from HCs in this study, whereas MD and b-tensor encoding metrics were more sensitive to HIV-related microstructural changes.

There are several limitations that should be acknowledged. First, this study is an exploratory cross-sectional pilot investigation with a limited sample size, which constrains statistical power and heightens the likelihood of both type I and type II errors. We present unadjusted *p*-values alongside effect sizes, and the findings should be regarded as hypothesis-generating rather than confirmatory.

Second, while the cognitive scores were norm-adjusted for age and education, the imaging metrics were not explicitly adjusted for these variables, potentially introducing residual demographic influences on the dMRI metrics. Furthermore, the observed cognitive differences between PWH and HCs cannot be attributed solely to HIV-driven factors, as other factors such as demographic variability, comorbidities, or lifestyle-related factors may influence cognitive performance.

Third, despite being age-matched, the PWH and HC groups differed in the proportions of male and female participants (roughly 25% in each group are female) and in the proportions of participants from different races, which could lead to residual confounding. Additional factors, such as substance use, cardiovascular risk, or other comorbidities, may have an impact on both dMRI and cognitive performance.

Fourth, while tensor-valued dMRI offers improved sensitivity to microstructural characteristics compared to standard DTI and MKI metrics, the biological specificity of these measures remains constrained, and the differences observed may reflect multiple underlying processes, including microstructural organization, extracellular free water content, or inflammatory changes.

Finally, the pattern of cognitive differences observed in this cohort (e.g., memory, learning, and fluency) does not fully align with the cognitive domains most commonly affected in HIV-associated neurocognitive impairment, which typically involves executive function, processing speed, and visuospatial abilities [64,65,66,67]. Therefore, within a specific clinical cohort (i.e., PWH), these factors may limit the generalizability of the findings. Larger and more diverse studies will be required to confirm these preliminary findings and better understand the role of tensor-valued dMRI metrics in characterizing brain changes and their cognitive correlates.

## 5. Conclusions

This study shows that tensor-valued dMRI is highly sensitive in detecting microstructural changes in major brain networks in cART-treated PWH. Reductions in µFA and MKa, and conventional FA and MK, indicate that white matter injury persists even after the cART treatment. PWH demonstrated deficits in learning, language, memory, and overall cognition, but attention and executive function appeared to be relatively intact. Notably, significant interactions between dMRI metrics and HIV status, particularly those involving MKi and attention, indicate altered relationships between tissue microstructure and cognition in PWH, possibly reflecting compensatory mechanisms or increased heterogeneity in cognitive performance across networks. These preliminary findings demonstrate the utility of tensor-valued dMRI as a sensitive biomarker of neurocognitive vulnerability in PWH. Future longitudinal multimodal studies with a larger sample size, incorporating tensor-valued dMRI, are required to characterize the progression of microstructural abnormalities and inform early intervention strategies for HIV-associated cognitive decline.

## Figures and Tables

**Table 1:** Subject Demographics and Clinical Characteristics.

Characteristics		PWH (n = 24)	HC (n = 31)	*p*-Value
**Age** **, mean ± SD**		55 ± 10	55 ± 15	0.947
**Sex, n**				0.443
	Female	7	7	
	Male	17	24	
**Ethnicity, n**				0.847
	Hispanic or Latino	0	2	
	Not Hispanic or Latino	23	29	
	Other	1	0	
**Race, n**				**<0.001**
	Caucasian	16	27	
	Black African American	6	4	
	Other	1	0	
	Missing	1	0	
**Education, n**				**<0.001**
	≤12 Years	4	1	
	>12 Years	20	30	
**Time since HIV diagnosis**				
**CD4+ T-cell count, cell/mm^3^, mean ± SD**		728 ± 288	NA	NA
**Viral load copies/mL, mean ± SD**		7 ± 13	NA	NA
**ARV drug class**				
	NRTI	4	NA	NA
	NNRTI	3	NA	NA
	PI	16	NA	NA

Note: Significant *p*-values are shown in bold. n: number of subjects, SD: standard deviation, ARV: Approved antiretroviral, NRTI: Nucleoside/Nucleotide Reverse Transcriptase Inhibitor, NNRTI: Non-Nucleoside Reverse Transcriptase Inhibitor, PI: Protease inhibitor, NA: Not applicable.

**Table 2:** Spearman correlations between MRI metrics in functionally defined ROIs and cognitive domain scores.

Network	Cohort		µFA vs. EXE	MKi vs. EXE	MKa vs. EXE	MD vs. EXE	µFA vs. ATT	MKi vs. ATT	MKa vs. ATT	MD vs. ATT	MKi vs. SPE
**DMN** **_D**	HC	*r (p)*	0.25 (0.185)	0.15 (0.419)	0.30 (0.113)	−0.21 (0.267)	**0.49 (0.006)**	−0.07 (0.698)	**0.51 (0.004)**	**−0.48 (0.005)**	0.11 (0.612)
		*d*	0.52	0.30	0.63	−0.43	1.12	−0.14	1.19	−1.09	0.22
	PWH	*r (p)*	**0.51 (0.016)**	**−0.56 (0.006)**	**0.52 (0.014)**	**−0.54 (0.016)**	0.23 (0.298)	**−0.53 (0.012)**	0.34 (0.119)	−0.09 (0.738)	**−0.62 (0.002)**
		*d*	1.19	−1.35	1.22	−1.28	0.47	−1.25	0.72	−0.18	−1.58
**DMN_V**	HC	*r (p)*	0.18 (0.348)	0.21 (0.266)	0.19 (0.314)	−0.07 (0.911)	**0.44 (0.016)**	−0.12 (0.528)	**0.40 (0.029)**	−0.34 (0.051)	0.12 (0.528)
		*d*	0.37	0.43	0.39	−0.14	0.98	−0.24	0.87	−0.72	0.24
	PWH	*r (p)*	**0.49 (0.020)**	−0.33 (0.129)	**0.43 (0.048)**	−0.25 (0.260)	0.21 (0.353)	−0.28 (0.210)	0.34 (0.119)	−0.10 (0.629)	**−0.62 (0.002)**
		*d*	1.12	−0.7	0.95	−0.52	0.43	−0.58	0.72	−0.20	−1.58
**ECN_L**	HC	*r (p)*	0.16 (0.391)	0.23 (0.222)	0.25 (0.181)	−0.03 (0.884)	**0.37 (0.046)**	0.19 (0.323)	**0.46 (0.011)**	−0.34 (0.066)	0.05 (0.867)
		*d*	0.32	0.47	0.52	−0.06	0.80	0.39	1.04	−0.72	0.1
	PWH	*r (p)*	**0.49 (0.022)**	−0.31 (0.161)	0.32 (0.152)	−0.33 (0.094)	0.22 (0.331)	−0.34 (0.122)	0.24 (0.287)	−0.11 (0.611)	−0.37 (0.088)
		*d*	1.12	−0.65	0.68	−0.70	0.45	−0.72	0.49	−0.22	−0.8
**ECN_R**	HC	*r (p)*	0.21 (0.273)	0.19 (0.315)	0.19 (0.315)	−0.20 (0.290)	**0.52 (0.003)**	0.06 (0.769)	**0.50 (0.005)**	**−0.42 (0.021)**	0.18 (0.340)
		*d*	0.43	0.39	0.39	−0.41	1.22	0.12	1.15	−0.93	0.37
	PWH	*r (p)*	**0.48 (0.023)**	**−0.43 (0.048)**	**0.43 (0.043)**	**−0.49 (0.019)**	0.26 (0.245)	−0.39 (0.078)	0.30 (0.178)	−0.03 (0.736)	−0.44 (0.056)
		*d*	1.09	−0.95	0.95	−1.12	0.54	−0.85	0.63	−0.06	−0.98
**SN_A**	HC	*r (p)*	0.17 (0.373)	0.19 (0.304)	0.09 (0.620)	−0.16 (0.465)	**0.49 (0.006)**	−0.00 (0.993)	**0.46 (0.010)**	**−0.50 (0.005)**	0.18 (0.340)
		*d*	0.35	0.39	0.18	−0.32	1.12	0.00	1.04	−1.15	0.37
	PWH	*r (p)*	**0.43 (0.046)**	**−0.44 (0.038)**	**0.48 (0.023)**	**−0.48 (0.016)**	0.15 (0.513)	−0.39 (0.074)	0.29 (0.187)	−0.04 (0.832)	**−0.50 (0.019)**
		*d*	0.95	−0.98	1.09	−1.09	0.30	−0.85	0.61	−0.08	−1.15
**SN_P**	HC	*r (p)*	0.12 (0.511)	0.23 (0.2170)	0.12 (0.540)	−0.16 (0.400)	**0.42 (0.022)**	0.00 (0.984)	**0.43 (0.018)**	**−0.44 (0.015)**	−0.01 (0.977)
		*d*	0.24	0.47	0.24	−0.32	0.93	0.00	0.95	−0.98	−0.02
	PWH	*r (p)*	0.26 (0.236)	−0.23 (0.310)	0.23 (0.301)	−0.29 (0.191)	0.15 (0.513)	0.00 (0.987)	0.12 (0.606)	−0.26 (0.097)	−0.32 (0.140)
		*d*	0.54	−0.47	0.47	−0.61	0.30	0.00	0.24	−0.54	−0.68

Note: EXE: executive function; ATT: Attention; SPE: Processing speed. Only the MRI metrics and cognitive domain that exhibit most significant associations are shown.

**Table 3:** An ordinary least squares linear regression model to measure the effects of dMRI metrics in functionally defined brain networks, HIV status, and their interactions on cognitive scores.

Cognition	Effect	Estimate	Std. Error	*t* Value	Cohen’s *d*	*Pr* (>|*t*|)
EXE	MKi DMN_D	2.2	5.6	0.39	0.10	0.696
	HIV Status	10.99	4.4	2.5	0.67	**0.016**
	HIV × MKi DMN_D	−21.65	8.84	−2.45	−0.65	**0.018**
EXE	ECN_R	6.66	4.40	1.51	0.40	0.136
	HIV Status	5.72	2.54	2.25	0.60	**0.029**
	HIV × MKi ECN_R	−11.29	5.09	−2.22	−0.59	**0.031**
ATT	ECN_L	2.55	1.65	1.55	0.41	0.128
	HIV Status	2.10	0.97	2.18	0.58	**0.034**
	HIV × MKi ECN_L	−4.17	1.95	−2.14	−0.57	**0.037**
ATT	ECN_L	2.55	1.65	1.55	0.41	0.128
	HIV Status	2.10	0.97	2.18	0.58	**0.034**
	HIV × MKi ECN_L	−4.17	1.95	−2.14	−0.57	**0.037**
ATT	ECN_L	3.86	1.13	3.43	0.92	**0.001**
	HIV Status	3.39	0.94	3.60	0.96	**0.001**
	HIV × MKt ECN_L	−4.53	1.29	−3.51	−0.94	**0.001**
ATT	ECN_R	4.07	1.36	3.00	0.8	**0.004**
	HIV Status	3.89	1.25	3.12	0.83	**0.003**
	HIV × MKt ECN_R	−5.05	1.65	−3.06	−0.82	**0.004**
ATT	ECN_R	−5.56	2.05	−2.71	−0.72	**0.009**
	HIV Status	−4.53	2.30	−1.97	−0.53	0.055
	HIV × MD ECN_R	6.06	2.98	2.03	0.54	**0.047**
ATT	SN_A	−5.98	1.96	−3.06	−0.82	**0.004**
	HIV Status	−5.82	2.47	−2.36	−0.63	**0.022**
	HIV × MD SN_A	7.13	2.93	2.43	0.65	**0.019**
ATT	SN_A	7.34	2.21	3.32	0.89	**0.002**
	HIV Status	2.87	1.31	2.20	0.59	**0.033**
	HIV × μFA SN_A	−6.55	3.17	−2.07	−0.55	**0.044**
ATT	SN_A	26.01	8.94	2.91	0.78	**0.005**
	HIV Status	5.77	2.37	2.44	0.65	**0.019**
	HIV × FA SN_A	−28.81	12.09	−2.38	−0.63	**0.021**
SPE	DMN_D	4.18	4.85	0.86	0.23	0.393
	HIV Status	11.69	3.81	3.07	0.82	**0.003**
	HIV × MKi DMN_D	−23.77	7.67	−3.10	−0.83	**0.003**
SPE	ECN_R	5.24	3.87	1.35	0.36	0.182
	HIV Status	4.93	2.23	2.21	0.59	**0.032**
	HIV × MKi ECN_R	−10.40	4.48	−2.32	−0.62	**0.024**
SPE	SN_A	9.52	4.82	1.98	0.53	0.054
	HIV_Status	12.52	4.89	2.56	0.68	**0.013**
	HIV × MKi SN_A	−25.62	9.80	−2.62	−0.7	**0.012**
LAN	SN_A	4.15	5.32	0.78	0.21	0.439
	HIV_Status	12.61	5.40	2.34	0.63	**0.024**
	HIV × MKi SN_A	−26.54	10.82	−2.45	−0.65	**0.018**

Note: EXE: executive function, ATT: Attention, LAN: Language, SPE: Processing speed.

## Data Availability

Anonymized data will be made available upon reasonable request, pending approval from the appropriate institutional review board.

## Abbreviations

The following abbreviations are used in this manuscript:

PWH	People with HIV
cART	Combination antiretroviral therapy
ARV	Approved antiretroviral
NRTI	Nucleoside/Nucleotide Reverse Transcriptase Inhibitor
NNRTI	Non-Nucleoside Reverse Transcriptase Inhibitor
PI	Protease inhibitor
SD	Standard deviation
dMRI	Diffusion magnetic resonance imaging
DTI	Diffusion tensor imaging
FA	Fractional anisotropy
MD	Mean diffusivity
DKI	Diffusion kurtosis imaging
MK	Mean Kurtosis
MKa	Anisotropic mean kurtosis
MKi	Isotropic mean kurtosis
MKt	Total mean kurtosis
µFA	microscopic fractional anisotropy
ROI	Region of interest
RSRB	Institutional Research Subjects Review Board
CalCAP	California Computerized Assessment Package
T1w	T1-weighted
MPRAGE	Magnetization-prepared rapid acquisition gradient-echo
TI	Inversion time
TE	Echo time
TR	Repetition time
GRAPPA	GeneRalized Autocalibrating Partial Parallel Acquisition
FOV	Field of view
DWI	Diffusion weighted imaging
FWF	Free waveform
STE	Spherical tensor encoding
LTE	Linear tensor encoding
FSL	FMRIB Software Library
ANTs	Advanced Normalization Tools
BET	Brain extraction tool
FAST	FMRIB’s Automated Segmentation Tool
FIRST	FMRIB’s Integrated Registration and Segmentation Tool
FLIRT	FMRIB’s Linear Image Registration Tool
FNIRT	FMRIB’s Nonlinear Image Registration Tool
DMN_D	Dorsal Default Mode Network
DMN_V	Ventral Default Mode Network
ECN_L	Left Executive Control Network
ECN_R	Right Executive Control Network
SN_A	Anterior Salience Network
SN_P	Posterior Salience Network
Cog	Cognitive score
OLS	Ordinary least square
OVERALL	Total cognitive score
LEA	Learning
LAN	Language
MEM	Memory
EXE	Executive function
SPE	Processing speed
MOT	Motor control
